# Supplementary material for: Analysis of the protein composition of the spindle pole body during sporulation in Ashbya gossypii
Source: PLoS One. 2019 Oct 3;14(10):e0223374. doi: 10.1371/journal.pone.0223374 (PMC6776394; doi:10.1371/journal.pone.0223374)
Supplement: S1 Fig — (PDF) [file pone.0223374.s001.pdf]

S1 Figure: Growth assays of strain carrying a single labeled SPB-component

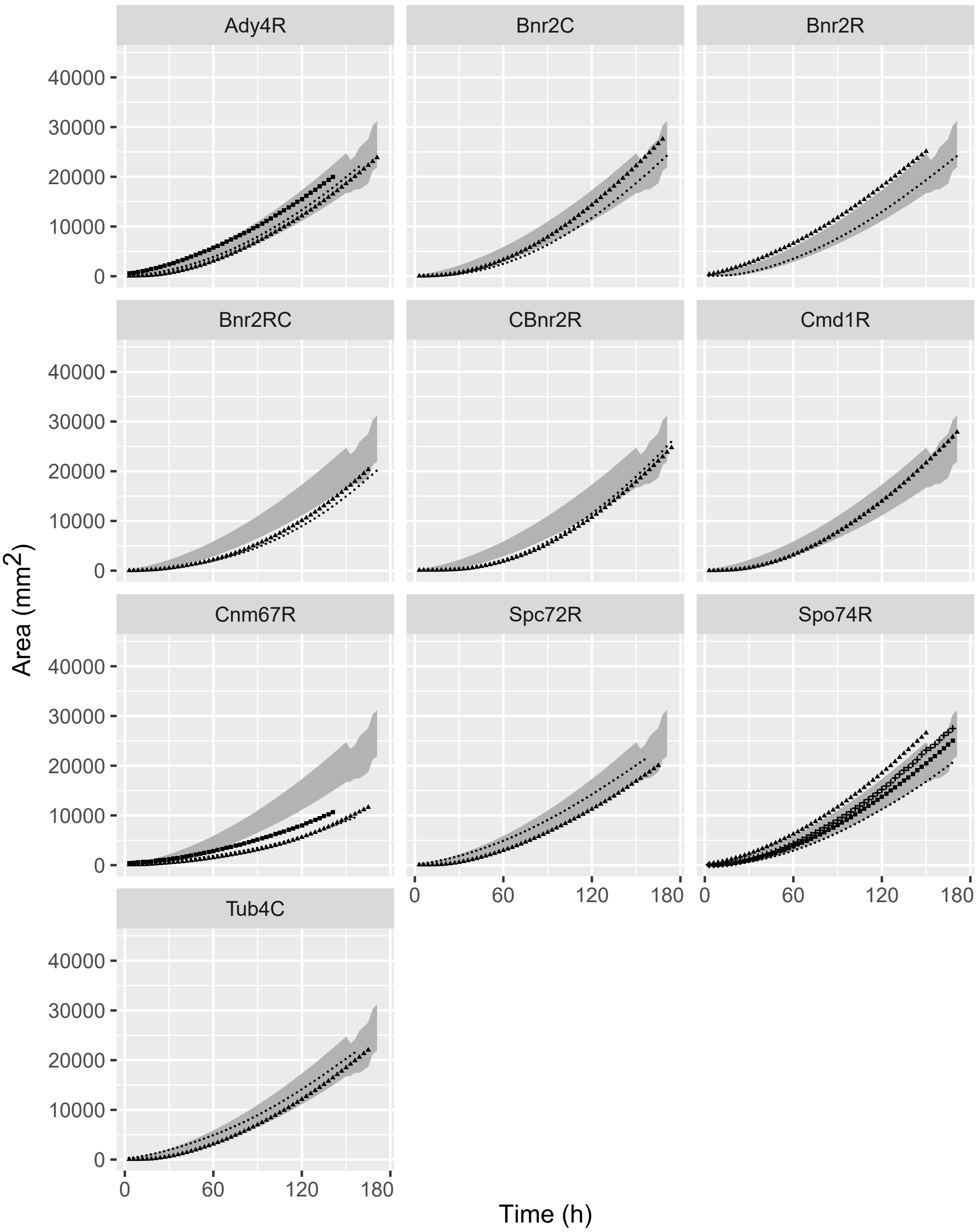

S1 Figure: Radial growth of strains carrying a single labeled SPB/SOP-component. The graph represents the area of the mycelium growing (in mm2) measured every 3 hours over the indicated time frame. The gray area represents the variations of at least 6 measured wildtype controls.
