# Supplementary material for: Analysis of the protein composition of the spindle pole body during sporulation in Ashbya gossypii
Source: PLoS One. 2019 Oct 3;14(10):e0223374. doi: 10.1371/journal.pone.0223374 (PMC6776394; doi:10.1371/journal.pone.0223374)
Supplement: Attachment — Submitted filename: Response to Reviewers.docx [file pone.0223374.s003.docx]

Reviewer comment:

-line 232-237: in Kemper et al. the FRET is described between GFP and RFP, CFP and YFP are mentioned only in the methods section. This is rather confusing and does not help very much in the discussion of why there are differences between the FRET measurements. I suggest the authors repeat the measurements with the original strains and their current analysis setup and include the data for comparison here, also because in the former publication no images of the FRET analysis were shown.

*Answer:*

*The statement of the reviewer is correct that there is a confusing, false statement in Kemper et al. (2011). The fusions used for the experiments in this study were yEGFP fused to AgBnr2 and tdtomato fused to Cnm67. We included measurements with these original strains in the data and also exchanged the images of the AgCnm67 fusion to the tdtomato version.*

Reviewer comment:

-line 240-250: initially, CBnr2R was categorized in the non-FRET group with frequently FRET positive signals. In the last sentence of the section, a positive FRET is claimed for the inactive form of CBnr2R, exactly as observed? Due to the high signal variation, this seems to be an over-interpretation of the data. Clearly more work is necessary to show clearly that Bnr2 shows self-interactions in its inactive form.

*Answer:*

*We have changed the statement in the text and rewrote the paragraph in the discussion about AgBnr2 self interaction.*

Reviewer comment:

-line 261-263: very confusing statements: both grow similar or one faster than wt? in the Figure, the left strain seems to grow faster, however this one contains Spo74. Also figure 4b shows images and 4c the growth curves.

*Answer:*

*The confusion here is caused because the text refers to an earlier version of the figure, which we have changed afterward. We have to apologize for overlooking that. We changed the text to “Both strains grew similar to control strains without the labeled proteins (Figure 4C) during the first 90 hours of growth. The strain with both labels, but with the SPO74 gene (left graph) grew even faster in the later growth phase.”*

Reviewer comment:

-line 321-323: if the authors do not fully trust the Y2H measurements of Kemper et al, why are these same Y2H interactions the main reason for the placement of the N-terminus of Bnr2 towards the IL1 in the model of figure 7? Maybe some more FRET measurements with N-terminal fusions are necessary to clarify this point and to have more support for the model.

*Answer:*

*We including the measurements with AgCnm67 fused to a different fluorophore, which now yield significant FRETR values. This resolves the discrepancy between the old FRET-data that did not display interaction and the 2-hybrid data. We have changed the text accordingly.*

Reviewer comment:

-line 325-327: in the citation Kemper et al., I could only find the usage of RFP and GFP for FRET measurements and only the interaction between Bnr2 and Cnm67 was tested by FRET. FRET data of Bnr2 with spo21 I could not find. Moreover, the FRET data of Kemper et al between Cnm67 and the C-terminus of Bnr2 contradicts the model in figure 7, that shows a placement of the C-terminus of Bnr2 that should not result in a FRET signal together with Cnm67.

*Answer:*

*We have changed the model to integrate the novel measurements of AgCnm67 and AgBnr2. From our data a conclusion about the orientation of AgBnr2 within the SPB is not possible, in addition it is, in contrast to the main SPC components also probably not static, but dynamic. We changed the discussion accordingly. The reviewer is also correct with AgSpo21. Kemper et al. contains colocalization-data, but no FRET measurements. This was confused with the AgSPC42-FRET measurements in the same paper. We removed all wrong references from the manuscript.*

Reviewer comment:

-line 364-351: too much speculation, see also the comment on Cnm67 FRET results.

Minor points:

*Answer:*

*We removed the parts containing speculation from the discussion and included discussion of the novel Cnm67 measurements.*

Reviewer comment:

-the FRET measurements have to be described in more detail. There are several crucial information that the reader is supposed to look after in diverse publications. These should be presented in the methods section as well as a good description of the adaptations that have been performed to the FRETSCAL algorithm. This will be important for everybody doing similar measurements in other fungi.

*Answer:*

*We have included a more detailed description of the FRET-measurements and a more detailed description of the analysis procedure.*

Reviewer comment:

- line 218-222: is there an estimation of how much of a mycelium was producing spores at a later time point? This information might be helpful to interpret the FRET data.

*Answer:*

*This is unfortunately a major problem in our system, which, so far we could not resolve. The fact that we might have looked at non sporulating SPBs in many cases is probably also responsible for the high variability in our measurements. We have undertaken many attempts to quantify sporulation reliably. Even though, some methods have been used before by other groups, none of these methods yields data that results in comparable numbers, even if the same culture is quantified multiple times, in our hands. In addition, there is another difficulty with quantifying sporulation under the microscope. During our measurements we realized that light seems to have a negative effect on sporulation. We have already looked deeper into that and identified potential factors involved in that regulation, which we will further investigate.*

Reviewer comment:

For the non-expert it would be helpful to more fully explain the defects in actin and/or chitin distribution seen in the spores of the Agmpc54∆ and Agady4∆ mutants. As only one cell is shown, it is not so clear how the mutants are abnormal. For Agmpc54∆, the authors state that similar defects were seen in mutants of rho1 and other actin-associated genes. I assume this refers to the elongated spore phenotype and not necessarily the effects on the distribution of actin, chitin, etc.? It appears in the earlier paper that not all of the mutations that caused lengthening of the spores produced the same effects on chitin distribution. Does Agmpc54∆ resemble a specific mutant? This discussion should be clarified.

*Answer:*

*We described in more detail, which phenotypes are similar to which other mutant and which phenotypes differ from the ones described before.*

Reviewer comment:

I may be overlooking it, but I do not see in the Methods a description of how the CFW/DiOC6/Eosin Y stainings were performed.

*Answer:*

*This information was indeed missing. The staining is described in detail in Lickfeld et al. (2012). We have added a statement and the reference to the methods section.*

Reviewer comment:

As part of their data collection for the FRET measurements, the authors should have generated measurements of the fluorescence intensity of mRuby fusions to multiple different SPB components. These values could be used to determine the relative stoichiometries of the different components. It would be of interest to compare these values to stoichiometries reported for the S. cerevisiae SPB.

*Answer:*

*We have integrated the data into Table 2 and added a paragraph discussing differences to Saccharomyces cerevisiae. In the discussion we focused on the components that were expressed from their own protomer and for which we could find comparable data in the literature.*

Reviewer comment:

Based on the studies in S. cerevisiae, where loss of either SPO74 or SPO21 causes complete absence of the MOP, it is surprising that AgSPO74 is important for AgBnr2 recruitment to the SOP but that AgSPO21 is not (the latter result is reported in an earlier paper from the Schmitz lab). It would be nice to revisit this second result here, but at a minimum this difference should be discussed.

*Answer:*

*We added a paragraph discussing this to the manuscript.*
