# Supplementary material for: Analysis of the protein composition of the spindle pole body during sporulation in Ashbya gossypii
Source: PLoS One. 2019 Oct 3;14(10):e0223374. doi: 10.1371/journal.pone.0223374 (PMC6776394; doi:10.1371/journal.pone.0223374)
Supplement: Attachment — Submitted filename: Response to Reviewers.docx [file pone.0223374.s004.docx]

Reviewer comment:

-Line 265-266: Figure 4A shows statistics, Figure 4B the images, the text is not correct here.

*We have changed the text to:*

“Figure 4 shows the statistics (Figure 4A) together with some sample images (Figure 4B) and the growth speed of the strains (Figure 4C).”

*Which now fits to the labeling in the figure.*

Reviewer comment:

- Line 366: "...actin does not seem to play a role in this yeast." Please provide a reference for this statement.

*We have added the reference:*

*“Taxis C, Maeder C, Reber S, Rathfelder N, Miura K, Greger K, Stelzer EH, Knop M. Dynamic organization of the actin cytoskeleton during meiosis and spore formation in budding yeast. Traffic. 2006;7(12):1628-42. PubMed PMID: 17118118.”*
